# Synovial hemangioma of the knee joint in a 12-year-old boy: a case report

**DOI:** 10.1186/1752-1947-4-105

**Published:** 2010-04-12

**Authors:** Anosheh Vakil-Adli, Shahin Zandieh, Josef Hochreiter, Monika Huber, Peter Ritschl

**Affiliations:** 1 Department of Orthopaedic and Orthopaedic Surgery, St Vincent's Hospital, Seilerstätte 4, Linz, Austria; 2 Department of Radiology, Hanusch Hospital, Vienna, Austria; 3 Department of Pathology, Otto-Wagner Hospital, Vienna, Austria; 4 Orthopaedic Hospital Gersthof, Vienna, Austria

## Abstract

**Introduction:**

Synovial hemangioma is a rare condition and is frequently misdiagnosed, leading to a diagnostic delay of many years.

**Case presentation:**

We present a case of an atypical synovial hemangioma in a 12-year-old Caucasian boy with a diagnostic delay of 3 years.

**Conclusion:**

It is important to know that synovial hemangioma mostly affects the knee joint, showing recurrent bloody effusions without a history of trauma. If there are no intermittent effusions, the diagnosis will be even more difficult. In cases of nonspecific symptoms and longstanding knee pain the diagnosis of a synovial hemangioma should also be considered in order to avoid diagnostic delay. Magnetic resonance imaging is the main diagnostic tool to evaluate patients with synovial hemangioma, showing characteristic lace-like or linear patterns.

Angiography can identify feeder vessels and offers the possibility of embolisation in the same setting. Surgical excision, either done per arthroscopy or per arthrotomy, is recommended as soon as possible to avoid the risk of damage to the cartilage.

## Introduction

Hemangiomas of bone constitute 1% of all primary bone tumours. The soft tissue types are even less common and often arise in the skin and subcutaneous tissue. Muscle and synovial linings are less frequent sites of origin. Since the first case was described by Bouchut in 1856, fewer than 200 cases have been reported. Most cases have been the intra-articular and intermediate type of hemangio-hamartoma, another form of vascular tumour of the leg representing an arteriovenous malformation which involves the synovia and causes intra-articular bleeding. Only a few of these have been true synovial hemangioma [1,2].

Usually the patient presents with a history of recurrent atraumatic bloody effusions [2-4].

Nonspecific presentations are also common and may lead to a diagnostic delay of many years [5]. We present the case of an atypical synovial hemangioma of the knee joint, having no single bloody effusion. Treatment methods have varied in the past. Angiography can help to find some feeder vessels and embolisation can be done in the same session.

In the absence of specific vessels to embolise, surgical excision, either done per arthroscopy or per arthrotomy, is the treatment of choice.

## Case presentation

A 12-year-old Caucasian boy presented with a history of pain and swelling in his left knee joint for 3 years for which he had received no previous treatment. His physical examination revealed a soft, non-tender, palpable 3 × 4 cm mass on the medial aspect of his left knee. In full flexion the mass appeared more pronounced. He denied any history of trauma and there was no effusion in his knee, with the joint not showing any signs of instability; he also had a full range of knee motion and normal strength in the lower extremities. McMurray and Apley tests were negative. There was no difference in leg length and there were no cutaneous lesions. Laboratory tests, including a complete coagulation profile, were all within normal range and his medical, developmental and family histories were unremarkable.

**Figure 1:**
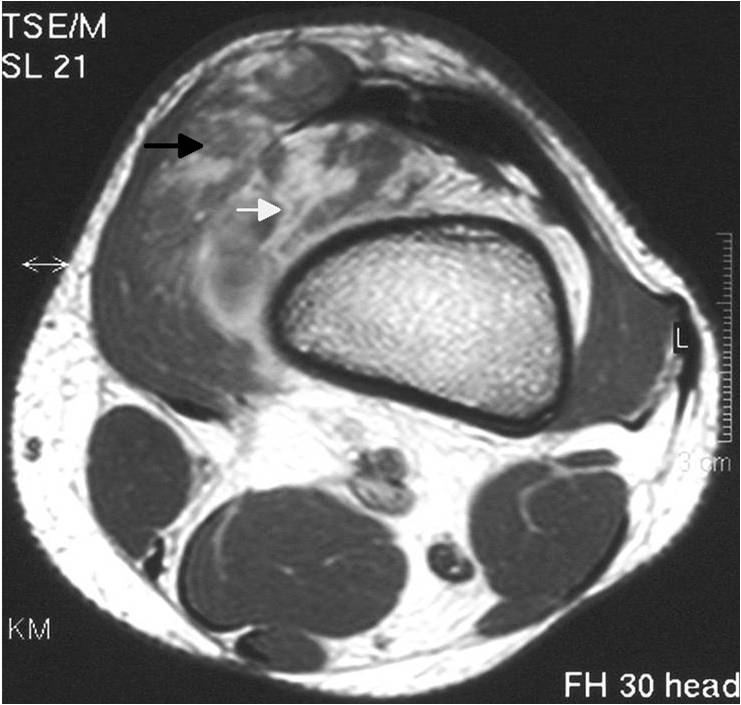
**Axial T1-weighted image after gadolinium administration demonstrates a mass of intermediate signal intensity with inhomogeneous enhancement in the suprapatellar pouch**. The tumour has an intra-articular (white arrow) and an extra-articular part (black arrow) and is not clearly distinguishable from the vastus medialis muscle.

**Figure 2:**
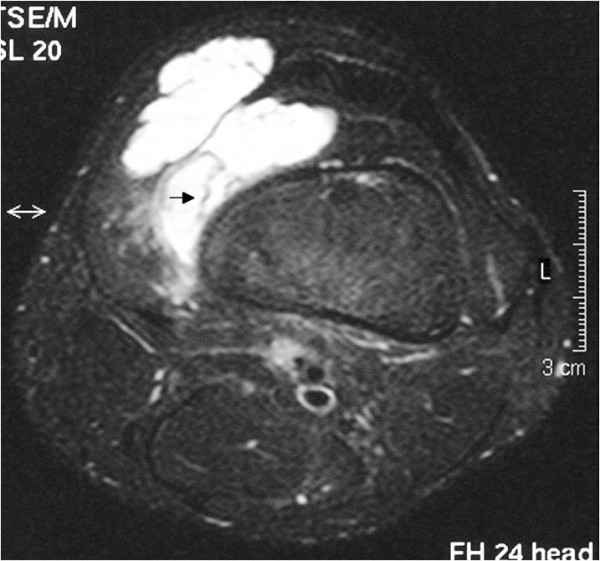
**Axial T2-weighted image with fat suppression technique shows the tumour with a high signal intensity in the exact size and extent**. A characteristic lace-like pattern (black arrow) and the tumour's extension into the vastus medialis muscle is seen.

**Figure 3:**
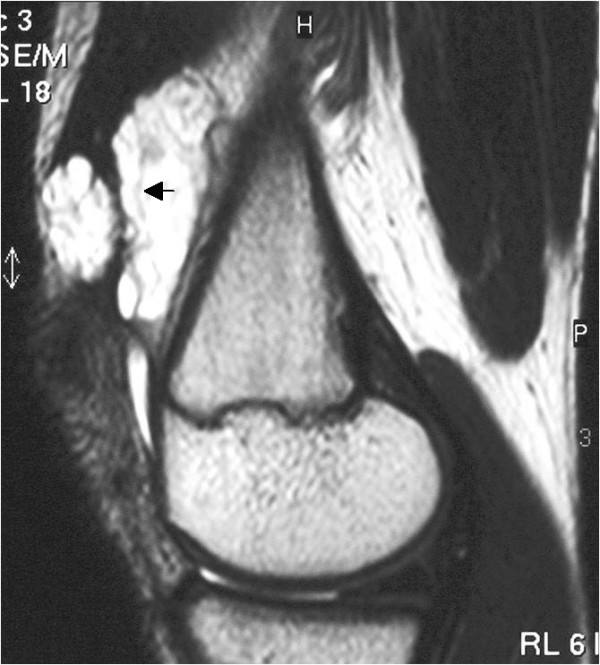
**Sagittal T2-weighted fat suppressed image of the left knee showing thin fibrofatty septae of low signal intensity within the lesion (black arrow)**.

Plain radiographs and magnetic resonance imaging (MRI) scans were obtained (Figures 1, 2 and 3). The anteroposterior and lateral radiographs of the left knee showed no abnormality, especially no signs of phleboliths but the MRI scan showed a well-defined mass located within the suprapatellar pouch, but infiltrating the vastus medialis muscle. The mass appeared lobulated in contour with internal septae. On T1-weighted images the lesion had a low or intermediate signal and was not clearly distinguishable from adjacent muscles (Figure 1). On T2-weighted images the mass had a signal intensity brighter than fat with thin fibrofatty septae of low-signal within the lesion (Figures 2 and 3). The differential diagnosis mainly included pigmented villonodular synovitis (PVNS) and synovial sarcoma.

**Figure 4:**
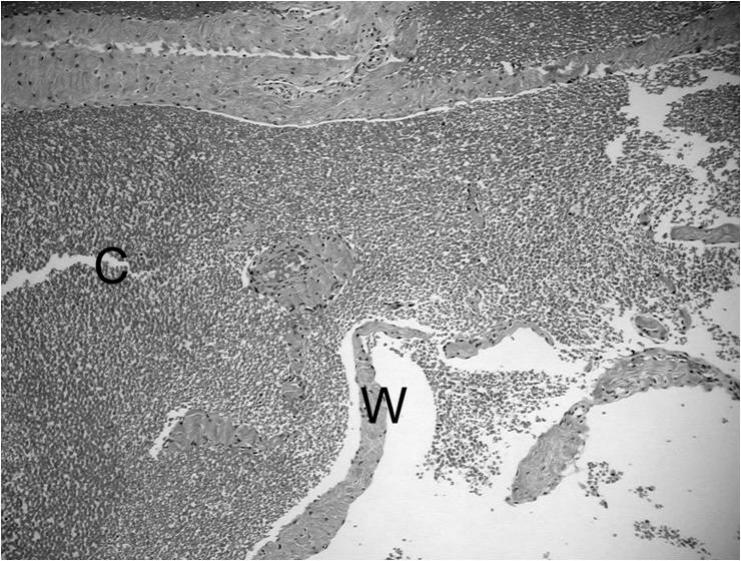
**Photomicrograph of the tumour, which is composed mainly of cavernous blood vessels**. Higher magnification shows the tumour tissue containing irregular large cavities (C) filled with blood and separated by thin walls (W).

Due to the differential diagnosis, an incisional biopsy was performed first, strictly according to the guidelines of orthopaedic tumour surgery. The biopsy specimen was 4 cm in diameter, measuring synovial tissue. An intraoperative frozen section showed a hemangioma with huge, cavernous spaces but also containing capillary vessels. Because of the diffuse extension of the hemangioma, angiography was done some days later in order to find some feeding arteries and to embolise them preoperatively in the same session. Angiographically, neither the hemangioma nor any feeding arteries could be visualized. Arthrotomy, through an anteromedial longitudinal skin incision, followed due to the diffuse extension of the tumour. The extra-articular and intra-articular masses were excised and the postoperative course was uneventful. The final histological evaluation confirmed a cavernous synovial hemangioma (Figure 4).

## Discussion

Synovial hemangiomas are frequently misdiagnosed leading to a diagnostic delay of many years; there are even reports of delays of up to 20 and 40 years [4-6].

Usually, a patient presents in childhood with a history of recurrent atraumatic painful bloody knee effusions [2-5]. These recurrent spontaneous hemarthroses of the knee joint and normal coagulation parameters should direct attention to the possibility of a synovial hemangioma. The clinical diagnosis is even more difficult without a history of intermittent effusions, as in our case. Plain films are often of poor diagnostic value because they are normal in over half of patients, and in other cases they show soft tissue density, suggesting joint effusion or a mass. They may contain phleboliths or amorphous calcifications; this is thought to be pathognomonic. In less than 5% of patients they show periosteal reaction, cortical destruction, osteoporosis, advanced maturation of the epiphyses and a discrepancy in leg length or even arthropathy simulating hemophilia [7]. Magnetic resonance imaging offers superior tissue contrast and is more accurate than computed tomography (CT) in defining the size and extent of a soft tissue lesion. It has become the main diagnostic method for the diagnosis and treatment planning of synovial lesions [8,9]. Synovial hemangioma usually shows intermediate signal intensity on T1-weighted images, although it may also contain areas of high signal intensity as in our case (Figure 1), due to intratumoral fat or blood products [10]. On T2-weighted images the lesion exhibits a high signal (brighter than fat) correlating with stagnant blood in vascular spaces (Figures 2 and 3) [8,10]. Both T1-weighted and T2-weighted images contain characteristic lace-like or linear patterns due to the histological structure of synovial hemangioma [2,8,11]. The high signal intensity after intravenous gadolinium administration can permit their differentiation from muscle. The use of contrast medium is indicated when there is an associated joint effusion, to better differentiate hemangioma from intra-articular fluid, which does not enhance. The differential diagnosis should include mainly PVNS and synovial sarcoma, other arthropathies (rheumatoid arthritis, juvenile chronic arthritis, hemophilic arthropathy, synovial osteochondromatosis or lipoma aborescens) usually being distinguished clinically or after MRI interpretation.

Angiography should be part of the diagnosis; it can define the size and location of the lesion and can identify feeder vessels or an associated arteriovenous malformation [1,2].

It must be performed early in cases of associated cutaneous hemangioma or abnormal varicosity, because these findings are indicative of a more general vascular abnormality [2].

In those instances selective embolisation of feeder vessels is an interesting alternative to surgery [12]. Angiography can fail by showing none or only part of the hemangioma in cases where the vascular channels are thrombosed, as in our case [13]. Synovial hemangiomas should be treated early because they can cause arthropathy, probably because of recurrent episodes of intra-articular bleeding and they can even infiltrate muscles, fat and cortical bone [9,14].

Treatment methods have varied in the past and include radiotherapy, open surgical resection, arthroscopic excision, arthroscopic ablation with a holmium, YAG laser, embolisation, and the use of sclerosing agents, cautery and freezing [1,15]. Some authors consider that arthroscopy is the gold standard in detecting and treating hemangioma of the knee [6]; it is reasonable if the tumour is focal or pedunculated and manageable in size [16]. In our case, with an intermediate type of synovial hemangioma (having an intra- and extra-articular part), arthrotomy was the only choice of treatment.

## Conclusion

Synovial hemangioma is a rare condition and mostly affects the knee joints. Recurrent bloody effusions without a history of trauma should alert the surgeon to this diagnosis. If there are no intermittent bloody effusions there may be a diagnostic delay of up to many years. For this reason a synovial hemangioma should also be considered in cases with nonspecific presentations and longstanding knee pain. If a synovial hemangioma is assumed, plain films are often of poor diagnostic value and magnetic resonance imaging is the main diagnostic tool to evaluate patients with a suspected synovial hemangioma. Angiography should also be part of the diagnostic approach as it can identify feeder vessels and offers the possibility of embolisation in the same setting. Several treatment methods have been proposed but in our opinion the treatment of choice is surgical excision; if the tumour is pedunculated and intra-articular, arthroscopy is the treatment of choice. If the synovial hemangioma is an intermediate type then arthrotomy should be performed. In any event, treatment should be initiated as early as possible to reduce the risk of damage to the cartilage.

## Consent

Written informed consent was obtained from the patient's parents for publication of this case report and accompanying images. A copy of the written consent is available for review by the Editor-in-Chief of this journal.

## Competing interests

The authors declare that they have no competing interests.

## Authors' contributions

SZ: study concept and design, patient care, drafting the manuscript AVA: study concept and design, patient care, data analysis, literature review, drafting and revising the manuscript JH: study concept and design, patient care, drafting the manuscript MH: data analysis, literature review and drafting the manuscript PR: study concept and design, patient care, drafting the manuscript. All authors read and approved the final manuscript and all participated in this work.
